# Intrauterine administration of autologous peripheral blood mononuclear cells regulates the endometrium estrogen and progesterone receptor expression: An RCT

**DOI:** 10.18502/ijrm.v21i4.13273

**Published:** 2023-05-08

**Authors:** Fattaneh Farifteh, Elham Fazeli, Seyedeh Zeinab Hosseini, Seyedeh Soheila Arefi, Ashraf Moini, Robabeh Taheripanah, Fatemeh Rouhollah, Mohammad Salehi, Ahmad Hosseini, Moncef Benkhalifa

**Affiliations:** ^1^Cellular and Molecular Biology Research Center, Shahid Beheshti University of Medical Sciences, Tehran, Iran.; ^2^Genetics and In Vitro Assisted Reproductive (GIVAR) Center, Erfan Hospital, Tehran, Iran.; ^3^Mehr Fertility Research Center , Guilan University of Medical Sciences , Rasht, Iran.; ^4^Department of Cellular and Molecular Sciences, Advanced Sciences and Technology, Tehran Medical Sciences, Islamic Azad University, Tehran, Iran.; ^5^Reproductive Biotechnology Research Center, Avicenna Research Institute, ACECR, Tehran, Iran.; ^6^Breast Disease Research Center, Tehran University of Medical Sciences, Tehran, Iran.; ^7^Department of Endocrinology and Female Infertility, Reproductive Biomedicine Research Center, Royan Institute for Reproductive Biomedicine, ACECR, Tehran, Iran.; ^8^Department of Gynecology and Obstetrics, Arash Women's Hospital, Tehran University of Medical Sciences, Tehran, Iran.; ^9^Infertility and Reproductive Health Research Center, Shahid Beheshti University of Medical Sciences, Tehran, Iran.; ^10^Department of Biotechnology, School of Medicine, Shahid Beheshti University of Medical Sciences, Tehran, Iran.; ^11^ART and Reproductive Genetics Department and PERITOX Laboratory, CURS, University Hospital and School of Medicine, Picardie University Jules Verne, Amiens Sud, France.

**Keywords:** Peripheral blood mononuclear cell, Estrogen receptors, Progesterone receptors.

## Abstract

**Background:**

Repeated implantation failure (RIF) affects 15% of women of reproductive age. There is a high endometrial expression of both estrogen receptors and progesterone receptors (PRs) during the window of implantation in women with RIF.

**Objective:**

To evaluate the effects of intrauterine administration of human peripheral blood mononuclear cells (PBMC) on estrogen receptor α (ERα) and PRs expression in the endometrium of women with RIF during the implantation window.

**Materials and Methods:**

This randomized clinical trial study was conducted on 22 women with RIF history from January 2018 to August 2019 in Erfan hospital, Tehran, Iran. Participantswere divided into 2 groups (PBMC-treated group [n = 11] and control group [n = 11]). Endometrial tissue samples were collected at the implantation window time, during the mid-secretory phase (luteinizing hormone surge +7 days) of each menstrual cycle. The quantitative real-time polymerase chain reaction technique was used to measure the mRNA levels of ERα and PRs isoforms (PR-A and PR-B) in endometrial tissues. Furthermore, the protein expression of ERα and PRs was investigated using immunohistochemical staining.

**Results:**

PBMC treatment significantly decreased the mRNA expression of endometrial ERα and PRs isoforms at the time of the implantation window (p < 0.001). Moreover, the endometrial ERα and PRs protein localization were significantly lower in PBMC-treated women compared with controls (p = 0.01, and p < 0.001 respectively).

**Conclusion:**

The intrauterine administration of PBMC decreased the endometrial ERα and PRs expression during the window of implantation in women with RIF. This local response to PBMC therapy could promote endometrial receptivity and embryo implantation.

## 1. Introduction

Despite the improvements in assisted reproductive technologies, the rates of embryo implantation and pregnancy remain suboptimal. Furthermore, a considerable number of couples have experienced repeated implantation failure (RIF). This situation affects 15% of women of reproductive age (1). In reproductive medicine, RIF refers to the failure of at least 3 consecutive fresh or frozen cycles in which 1 or 2 good-quality embryos were transferred (2). Among couples undergoing assisted reproduction treatment, RIF significantly increases the level of distress experienced by couples and also increases the cost of treatment (3).

Embryo-uterine cross talk synchronizations are a critical factor for successful blastocyst implantation. The uterine endometrium is permissive to the implanting blastocyst only during the “implantation window”. In humans, this timely event occurs at an interval of 4-6 days during the mid-luteal phase and is regulated by synergistic signals of the ovarian hormone's estrogen and progesterone (4). The function of progesterone and estrogen hormones is through their specific nuclear receptors that act as chromatin modifiers and ligand-activated transcription factors to control multiple** gene expressions (4). Estrogen receptors (ERs) have 2 isoforms, ERα and β, derived from 2 different genes including estrogen receptor *ESR1* and *ESR2* (5). Studies using ESR1 and ESR2 knockout female mice have displayed that ERα but not ERβ, plays an important role in blastocyst attachment to the endometrium (6). There are 2 progesterone receptors (PRs) isoforms, PR-A and PR-B. In contrast to ERs, the PRs isoforms are derived from a single gene (progesterone receptor [PGR]) by alternate transcription and translation initiation sites (5). Both ER and PR are downregulated at the time of implantation. It has been reported that the downregulation of both ER and PR during the window of implantation is an extremely important event, which influences the endometrial receptivity (4). Recent evidence has shown that estrogen and progesterone interact with the immune system during menstrual cycle and pregnancy, and their receptors are identified on immune cells (7-13).

Many studies have investigated local immune cells involved in implantation and are actively engaged in embryo implantation (3, 14-17). It has been shown that PBMC intrauterine administration increases several cytokines production, including interleukin (IL)-1α, IL-1β, and tumor necrosis factor (TNF) α in the endometrium, which has an essential role in uterine receptivity (18). Besides, it has been shown that PBMC derived from women in early pregnancy enhances the BeWo cell and murine blastocyst invasion (19). In mice, intrauterine administration of PBMC during the implantation window increases endometrial vascular endothelial growth factor and leukemia inhibitory factor gene expressions and implantation rate (15).

Several studies have reported that intrauterine administration of PBMC could improve implantation and pregnancy outcomes in women with RIF (3, 14, 16, 17, 20-22). The immunomodulation techniques could be a promising approach in the treatment of numerous RIF cases and in promoting in vitro fertilization (IVF) success rate (16). Therefore, this study was proposed to investigate the effects of intrauterine administration of human PBMC on ERs and PRs expression in the endometrium of women with RIF during the implantation window, to clarify the molecular mechanism of intrauterine administration of PBMC that can modulate endometrial receptivity and blastocyst implantation in women with RIF.

## 2. Materials and Methods

### Subjects

This randomized clinical trial study was conducted on 22 women who attended the Genetics and In Vitro Assisted Reproductive Center of Erfan hospital, Tehran, Iran for IVF treatment, from January 2018 to August 2019. Women with an RIF history who had primary infertility and had not received any steroidal hormone ≥ 2 months were included in this study. Infertile women having serially failed embryo implantation following at least 3 transfer cycles with good-quality embryos were selected for the study.

The main exclusion criteria were genetic disorders, history of repeated infectious diseases, endometriosis, and uterine anatomic abnormalities. The participants were randomized bya computer-generated random number table into 2 groups (PBMC-treated group and control group).

### Study design 

Participantsconsisted of infertile women in the natural cycle who underwent hysteroscopy as a diagnosis procedure before their infertility treatment.

Subjects were intended to the PBMC-treated group who received intrauterine administration of PBMC, 2 days before endometrial biopsy (n = 11) and the RIF control group undergoing endometrial biopsy without a previous intrauterine PBMC administration (n = 11).

### PBMC treatment protocol

The human PBMC preparation was performed as described previously (9). Briefly, a peripheral blood specimenwas collected 5 days before the mid-secretory endometrial biopsy. Then PBMC was separated using a ficoll-hypaque gradient (Histopaque, Sigma-Aldrich, USA) according to the manufacturer ${}^{{}^{'}}$ s instructions. Isolated PBMC (1 × 10^6^ cells/mL) was suspended in basic culture medium (Advanced Technologies Laboratory, La Verrière, France) consisting of growth factors, ILs, cytokines, mitotic factors, and corticoid-releasing hormone and incubated in 30 mm polystyrene tissue culture dishes for 48-72 hr at 37 C under atmosphere air and 5% CO_2_. 2 days before the endometrial biopsy, the cultured PBMC (2 × 10^7^ cells/200 μl in PBS) was slowly transferred into the women uterine cavity using an embryo transfer catheter (Cook Ltd, UK).

### Endometrial biopsy sampling

Endometrial biopsies were collected from all participants at the time of implantation window. In the current study, implantation window is defined as 7 days after luteinizing hormone surge (luteinizing hormone surge +7 days) in mid-secretory phase of each menstrual cycle. Endometrial tissue samples were obtained using a Pipelle sampler from the anterior wall of the uterine cavity, without cervical dilatation. The biopsy samples were divided into 2 portions. One part was prepared for immunohistochemicalanalysis, and the other part was frozen in liquid nitrogen and stored at -80 C until used for RNA extraction and quantitative polymerase chain reaction.

### RNA isolation and cDNA synthesis

The quantitative real-time polymerase chain reaction (qRT-PCR) technique was applied to determine the mRNA levels of *ESR1*, *PGR,* and *PGRB *(PRs B). Isolation of total RNA from endometrial samples was performed using a commercial kit (Gene All Biotechnology Co., South Korea) according to the manufacturer's instructions (23). In summary, samples were placed in Eppendorf tubes with 1.5 μl lysis buffer. Next, 4 μl RNA, 5 μl water, and 2 μl hexamer were added to each tube and placed in a thermocycler at 75 C for 5 min. Tubes were subsequently placed in an ice bath, then 1 μl of RT enzyme (200 u), 5 μl of 5 × RT buffer, 0.25 μl RNase inhibitor (10 u), and 3 μl dNTP (10 mM) were added to the tubes and placed in GeneMate thermocycler. The reverse transcription amplification program step was as follows: 10 min (25 C), 15 min (37 C), 45 min (42 C), and 10 min (72 C). The samples were placed in a cooler at 4 C overnight, after the reverse transcriptase reaction.**

### qRT-PCR

qRT-PCR analyses of *ESR1*, *PGR*, and *PGRB* mRNA were carried out using ABI StepOne Plus (Applied Biosystems). The sequences of gene-specific primers and annealing temperature are summarized in table I. Because the 2 PRs isoforms are expressed as a product of one gene (the PR-Asequence is included in the longer PR-B transcript), it is currently impossible to design primers for the detection of the PR-A mRNA alone by qRT-PCR (24). qRT-PCR reactions were performed in a final volume of 13 μl following the manufacturer's protocol for DNA Master SYBR Green I mix (Roche Applied Sciences). The reaction conditions: 2 min at 95 C, 5 sec at 95 C for denaturation, 30 sec at 60 C, 10 sec at 72 C for amplification, and 40 cycles of extension. PCR products were detected using a melting curve generation. All reactions were carried out in 3 replications. Glyceraldehyde-3-phosphate dehydrogenase gene expression was adjusted as the internal reference gene. The expression level of each target gene was normalized to the internal reference gene mRNA level. The relative gene expression level was measured using 2-ΔΔCt and presented as a fold change of a control sample.

### Immunohistochemistry

ERα and PR protein expression were evaluated using immunohistochemical staining. The endometrial samples were immediately fixed by 10% neutral buffered formalin overnight and next placed in paraffin wax. The endometrium samples were sectioned at a thickness of 5 μm and fixed with 4% paraformaldehyde in PBS for 20 min at 4 C. The PBS-washed sections were then incubated in 2N HCl at room temperature for 20 min. Then, 0.3% Triton X100 was applied for 30 min at room temperature for permeabilization. After washing, the samples were blocked using 10% normal goat serum in PBS at room temperature for 30 min. Primary mouse monoclonal anti-ERα (1:100 dilutions; Santa Cruse) and a mouse monoclonal anti-PR (1:100 dilutions; Santa Cruse) antibodies for detection of PR-A and PR-B were incubated with sections overnight in humidified a chamber at 4 C. The sections subsequently were incubated in goat anti-mouse IgG (FITC)-conjugated secondary antibodies (1;1000 dilutions; Abcam) in the darkness at 37 C for 1 hr. Sections were then mounted with propidium iodide (PI) (Sigma-Aldrich, USA) for nucleolus staining. Digital pictures were taken for each slide using fluorescence microscopy (Olympus BX51) at × 400 magnification and the fluorescent signals in the endometrium were evaluated. All tissue samples were analyzed in 3 replications. The fluorescent intensities of staining in endometrial cells were examined using Image J software (NIH, Bethesda, Maryland, USA), version 1.41 that assessed the mean pixel of fluorescence signals. All qualitative and quantitative assessment of samples was done by a single, blinded investigator.

The primary outcomes included the mRNA levels of endometrial ERα and PRs isoforms measured using the qRT-PCR and the secondary outcomes were the endometrial protein expression of ERα and PRs investigated using immunohistochemical staining in the implantation window time, during the mid-secretory phase (luteinizing hormone surge +7 days) of the menstrual cycle.

**Table 1:** Primer sequences used for qRT-PCR


**Gene**	**Sequence**	**Length**	**GC%**	**Tm**
***ESR1 F***	5 ' CAAGAGAAGTATTCAAGGACATAACG 3 '	26	38.46	58.10
***ESR1 R***	5 ' GTATCCCACCTTTCATCATTCCC 3 '	23	47.83	58.85
***PGR F***	5 ' GTGGTCTAAATCATTGCCAGG 3 '	21	47.62	56.94
***PGR R***	5 ' CTTTCATCCGCTGTTCATTTAG 3 '	22	40.91	55.80
***PGRB F***	5 ' CCTTGTTGTATTTGTGCGTGTGG3 '	23	47.83	61.09
***PGRB R***	5 ' TCCACTGCCCCCTCACTAAAAC 3 '	22	54.55	62.21
***GAPDH F***	5 ' CAACTACATGGTCTACATG 3 '	19	42.11	50.23
***GAPDH R***	5 ' CTCGCTCCTGGAAGATG 3 '	17	58.82	53.83
qRT-PCR: Quantitative real-time polymerase chain reaction, GC: Guanine cytosine, Tm: Temperature, *ESR1*: Estrogen receptor 1, *PGR*: Progesterone receptor, *PGRB*: Progesterone receptor B, *GAPDH*: Glyceraldehyde-3-phosphate dehydrogenase				

### Ethical considerations

This study had received prior approval from the Ethics Committee at Shahid Beheshti University of Medical Sciences, Tehran, Iran

 (Code: IR.SBMU.RETECH.REC.1395.402) and registered at IRCT

 website (last update: 2022-08-06). An informed consent

 form was obtained from each participant.

### Statistical analysis

In this study statistical analysis was conducted with Statistical Package for the Social Sciences, version 16.0, SPSS Inc, Chicago, Illinois, USA (SPSS) software. Data were expressed as means ± SD. Students *t* test analysis was performed to evaluate the difference between the data. A p-value of < 0.05 was regarded as statistically significant.

## 3. Results

**Figure 1:**
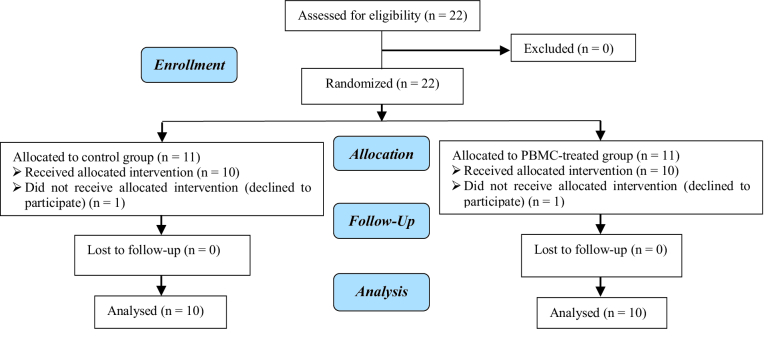
Study protocol. PBMC: Peripheral blood mononuclear cells.

A total of 22 women were defined into 2 groups: control (n = 11) and PBMC-treated group (n = 11). One participant in each group withdrew from the study due to personal reasons (Figure 1). The mean age of participants was 33.90 ± 2.71 yr. The baseline characteristics of women are presented in table II.

### mRNA expression levels of ESR1, PGR, and PGRB in the endometrium

**Figure 2:**
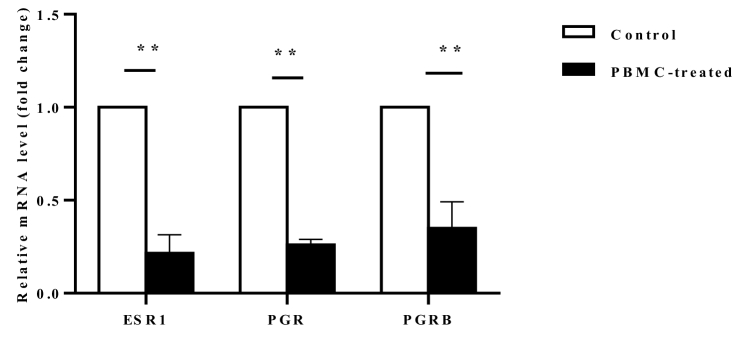
The mRNA expression of endometrial *ESRI* and *PGR* isoforms at the time of the implantation window of the menstrual cycle was performed using RT-qPCR assay. The relative expression levels were normalized to the *GAPDH* in the same sample. The relative expression levels of *ESR1*, *PGR,* and *PGRB* were significantly lower in PBMC-treated (N = 10) compared to the control group (N = 10) (**P < 0.01).

Intrauterine administration of human PBMC was administered with the objective of *ESR1*, *PGR,* and *PGRB* expression in the endometrium. Using qRT-PCR, we confirmed that relative expression levels of endometrial *ESRI* were significantly downregulated in PBMC-treated than control groups in the implantation window time of the menstrual cycle (p < 0.001). Moreover, we evaluated mRNA expression levels of 2 *PGR* isoforms by qRT-PCR. Our data confirmed that mRNA expression levels of *PGR* and *PGRB* were significantly lower in the PBMC-treated group endometrial tissues in comparison with control group (p < 0.001) (Figure 2).

### Cellular localization of ERα and PR proteins in the endometrium 

**Figure 3:**
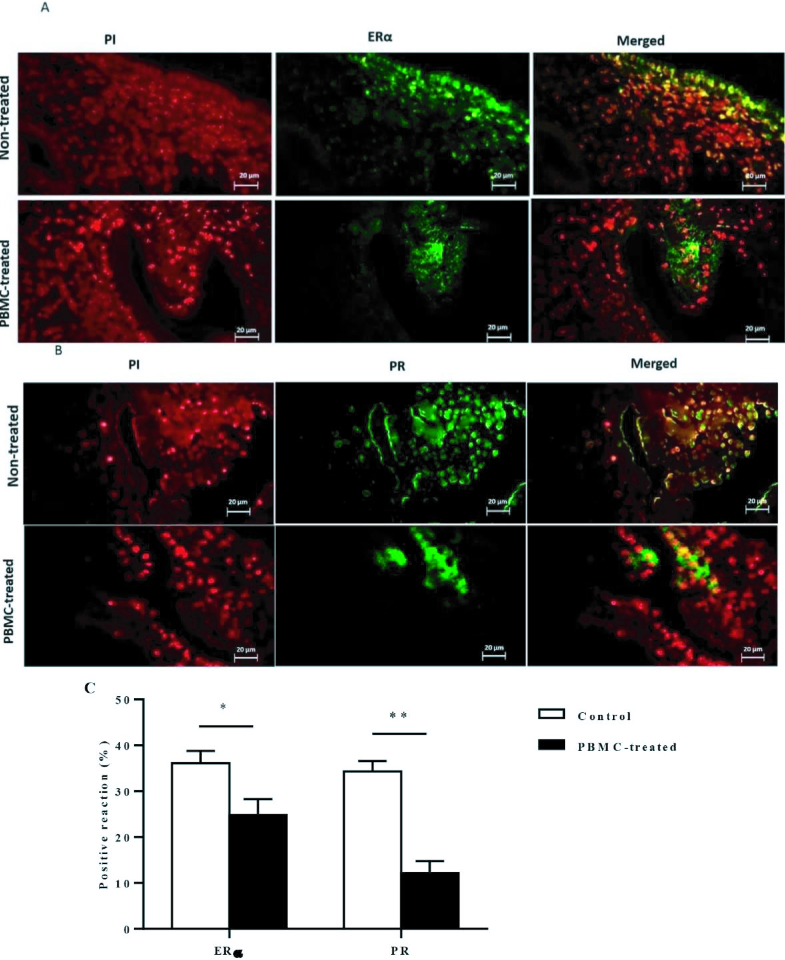
A, B) ERα and PR protein expression in human endometrium. Fluorescent immunohistochemical analysis of ERα and PR protein expression in the endometrial section was carried out. Cell nuclei were stained with PI (red). Then ERα and PR specific signal and PI were merged, showing that ERα protein localization in the endometrial cells. C) The endometrial ERα and PR protein expression were significantly decreased in PBMC-treated (N = 10) compared to control group (N = 10). Endometrial tissue samples of each participant were evaluated in triplicate (*P < 0.05, **P < 0.01). Magnification = × 400 (bar = 20 μm).

To assess the effects of intrauterine administration of human PBMC on ERα and PR-A and PR-B protein expression in endometrium during the implantation window of the menstrual cycle that were evaluated by immunohistochemistry (n = 20). Our data indicated that endometrial ERα protein localization was significantly decreased in PBMC-treated women than control women during the implantation window of the menstrual cycle (p = 0.01, Figure 3A). Moreover, immunohistochemical analysis of endometrial sections identified that on comparison with control women, those in PBMC-treated cycles showed significant decreases in endometrial PR-A and PR-B protein levels (p < 0.001, Figure 3B). Thereby, these results showed that PBMC treatment promotes endometrial receptivity among women who suffer from RIF (Figure 3C).

**Table 2:** Baseline characteristics of study subjects


	**Group**		
**Variables **	**PBMC-treated (n = 10)**	**Control (n = 10)**	**P-value**
	Age (yr)*	33.10 ± 2.80 (27-37)		34.70 ± 2.49 (30-38)	0.19
BMI (kg/m^2^)*	24.34 ± 3.12 (19.8-27.6)	24.63 ± 3.43 (20.4-28.9)	0.84
No. of previous implantation failure*	4.20 ± 3.11 (3-13)	4.30 ± 2.40 (3-11)	0.93
Data presented as Mean ± SD (minimum-maximum) *t* test, PBMC: Peripheral blood mononuclear cells, BMI: Body mass index			

## 4. Discussion

In this study we determine the effect of PBMC intrauterine administration on ERα and PRs expression in the endometrium of women with RIF. In the present study, qRT-PCR showed that relative expression levels of endometrial *ESRI* and *PGR* isoforms were significantly downregulated after PBMC intrauterine administration. In line with this, lower ERα, PR-A, and PR-B protein expressions in endometrium were recognized using the immunohistochemistry staining method in women treated with PBMC. These findings demonstrate that the intrauterine administration of PBMC may play an essential role in the endometrial receptivity and implantation process.

Successful blastocyst implantation is coordinately regulated by the interaction between embryo and adhesion molecules, cytokines, paracrine, and endocrine hormones in the endometrium (14). It has been shown poor endometrial receptivity causes unsuccessful blastocyst attachment in IVF cycles (4). RIF is a limiting factor in the success of IVF programs (16). It has been reported that endometrial receptivity decreased in women who suffered from RIF (25).

It is important to note that the dysregulation of immune mechanisms of feto-maternal dialogue can be a major cause of RIFs. The immunotolerance theory supports the view that CD56+ uterine NK cells are especially critical for successful embryo implantation (15). It has also recently been observed that intrauterine PBMC administration contributes to the production of several cytokines, including IL1, IL2, IL6, IL12, IL15, leukemia inhibitory factor, and TNFα which induce a T-helper1 dominantproinflammatory cytokines profile (16). It is also well-accepted that increased proinflammatory cytokines facilitate endometrial tissue remodeling for embryo implantation (26).

It has been shown that inflammatory cytokines exposure alters most protein and mRNA expression. The result of the present study also demonstrated a significant decrease in PR expression by PBMC treatment. In agreement with our findings, Grandi and co-workers reported that exposure to inflammatory cytokines TNFα and IL1β reduced the expression of PR mRNA and protein expression in the endometrial stromal cells of women with endometriosis (27).

The investigation of the endometrium of women with endometriosis showed chronic inflammatory states and progesterone resistance. It has been demonstrated that endometriosis implants contain high levels of proinflammatory cytokines, which upregulated nuclear factor kappa light-chain-enhancer of activated B cells (NF-kB) expression in endometrial cells and, NF-kB consequently, decreased PRs expression and progesterone function. NF-kB consists of a family of transcriptionfactors that regulategenesinvolvedinapoptosis, adhesion, and inflammation. In the current study, the reduction of progesterone RNA and protein expression in endometrial cells in women who suffer from RIF might contribute to the suppression of progesterone signaling by the inflammatory environment. Current theories suggest that PBMC stimulates proinflammatory cytokines production which increases NF-kB expression consequently decreases PRs expression. Furthermore, PBMC might directly decrease the level of both PR isoforms through epigenetic modifications (28). It has been demonstrated that the exposure of TNFα to endometriotic cells resultedin hypermethylation of the PR-B promoter, leadingto PR-B downregulation (29).

It is important to note that temporalandendocrinecontrol of ERα mRNA expression during the implantation window revealed its crucial roles in embryo implantation. High endometrial estrogen production can block the endometrial integrin αvβ3 molecules, a key factor in blastocyst-uterus attachments. Decreased integrin expression correlated with poor IVF success (30). To demonstrate how the PBMC intrauterine administration affects the endometrial ERα, it has been determined that in normal human endometrial epithelial cells there is an interaction between the NF-kB signaling pathways and ER expression (31). Furthermore, reciprocal inhibition of signaling between NF-kB and ER has been described in various in-vitro systems (32-34).

For example, in Triple Negative Breast Cancer Aspros have shown an interaction between ER and NF-kB signaling (35). In a reproductive context, it has been shown that in ectopic endometrial cells in the presence of estrogen, IL1β suppresses estrogen response element function and that there is also a mutual inhibition of estrogen on NF-kB signaling. Furthermore, it has been shown that NF-kB activation diminishes the response of endometrial stromal cells and Ishikawa cells to estrogen (36). Attributable to the activation of proinflamatory cytokine by PBMC treatment that involves NF-kB signaling, it can be one possible mechanism of ER downregulation in the current study.

It is also well-accepted that among some women with RIF, there is an inappropriate inflammatory environment in the uterine cavity (37). Clinical data have shown that the immunological environment of the blastocyst implantation site can be modulated by PBMC-derived cytokines (16). The association between intrauterine administration of PBMC and the subsequent increase in the success rate of embryo implantation in women with RIF is well established (37, 38). In this study, we demonstrated that immune therapy using autologous PBMC results in a significant decrease in protein and mRNA expression of ERα and PR in the endometrium of women with RIF. Downregulation of both ER and PR during the window of implantation was reported to be an extremely important event in ensuring endometrial receptivity (4). This reduction of ERα and PR in the endometrium of women with RIF may be one of the mechanisms that explain the significant increase in implantation and pregnancy rates following intrauterine administration of PBMC in previous studies. Further investigation is needed to delineate the molecular mechanism causing the downregulation of these steroid receptors (ERα and PR) post-intrauterine PBMC treatment. Like other clinical studies, our study was not without any limitations. The sample size of the current study was relatively small. So, our result can be provided by increasing the sample size in future studies. Therefore, more investigations are needed to clarify the underlying mechanisms of PBMC treatment on pregnancy outcomes.

## 5. Conclusion

In conclusion, we provide new evidence demonstrating that the intrauterine administration of PBMC decreased the endometrial ERα and PR expression during the window of implantation in women with RIF. This local response to PBMC therapy could promote endometrial receptivity and embryo implantation.

##  Conflict of Interest

The authors declare that there is no conflict of interest.
